# Time-Lapse Dynamics of the Mouse Oocyte Chromatin Organisation during Meiotic Resumption

**DOI:** 10.1155/2014/207357

**Published:** 2014-03-30

**Authors:** Martina Belli, Giulia Vigone, Valeria Merico, Carlo Alberto Redi, Silvia Garagna, Maurizio Zuccotti

**Affiliations:** ^1^Laboratorio di Biologia dello Sviluppo, Dipartimento di Biologia e Biotecnologie “Lazzaro Spallanzani”, Università degli Studi di Pavia, Via Ferrata 9, 27100 Pavia, Italy; ^2^Centro Ricerche di Medicina Rigenerativa, Fondazione IRCCS Policlinico San Matteo, 27100 Pavia, Italy; ^3^Centro di Ingegneria Tissutale, Università degli Studi di Pavia, 27100 Pavia, Italy; ^4^Sezione di Anatomia, Istologia ed Embriologia, Dipartimento di Scienze Biomediche, Biotecnologiche e Traslazionali (S.Bi.Bi.T.), Università degli Studi di Parma, Via Volturno 39, 43125 Parma, Italy

## Abstract

In the mammalian oocyte, distinct patterns of centromeres and pericentromeric heterochromatin localisation correlate with the gamete's developmental competence. Mouse antral oocytes display two main types of chromatin organisation: SN oocytes, with a ring of Hoechst-positive chromatin surrounding the nucleolus, and NSN oocytes lacking this ring. When matured to MII and fertilised, only SN oocytes develop beyond the 2-cell, and reach full term. To give detailed information on the dynamics of the SN or NSN chromatin during meiosis resumption, we performed a 9 hr time-lapse observation. The main significant differences recorded are: (1) reduction of the nuclear area only in SN oocytes; (2) ~17 min delay of GVBD in NSN oocytes; (3) chromatin condensation, after GVBD, in SN oocytes; (4) formation of 4-5 CHCs in SN oocytes; (5) increase of the perivitelline space, ~57 min later in NSN oocytes; (6) formation of a rosette-like disposition of CHCs, ~84 min later in SN oocytes; (7) appearance of the MI plate ~40 min later in NSN oocytes. Overall, we described a pathway of transition from the GV to the MII stage that is punctuated of discrete recordable events showing their specificity and occurring with different time kinetics in the two types of oocytes.

## 1. Introduction

In the nucleus of eukaryotic cells, chromosomes occupy distinct territories whose position may change during the cell cycle or cell differentiation [1–4]. Entire chromosomes, subchromosomal regions, and genes change their nuclear localisation during differentiation to acquire a cell-type-specific spatial organisation, contributing, as part of the epigenome, to the regulation of the cell functions [5–8]. Centromeres and pericentromeric constitutive heterochromatin (CHC) of mammalian cells tend to gather to form chromocenters. The degree of centromere clustering varies depending on the cell type, cell-cycle phase, or stage of differentiation [9–14]. Lineage-specific centromere associations into chromocenters have been reported during somatic [9, 15, 16], male [17], and female [18, 19] germ cell differentiation. Within the nucleus, the nucleolus is a major attractive compartment for heterochromatic regions such as inactive X-chromosome, regions enriched in repressed genes, and pericentromeric repeated sequences [20]. Altogether, these studies indicate that a specific nuclear localisation of these chromatin traits is required for a correct genome functioning at different cell-cycle phases and different stages of cell differentiation [21].

To this regard, the mammalian oocyte is a particularly intriguing cell model study as distinct patterns of centromeres and pericentromeric CHC localisation correlate with the gamete meiotic and developmental competence. When stained with Hoechst 33342 (Ho), a supravital fluorochrome that preferentially binds to the AT-rich regions of the genome, fully grown germinal vesicle (GV) mouse antral oocytes display two main different types of chromatin organisation: Surrounded Nucleolus (SN) oocytes, with a ring of Ho-positive chromatin surrounding the nucleolus and Nonsurrounded Nucleolus (NSN) oocytes, lacking the ring and with a more dispersed chromatin [22–24]. A bold of experimental evidence has shown that, following their isolation from the ovarian surface, SN and NSN antral oocytes display a different meiotic competence, with 82% or 45% of SN and NSN oocytes, respectively, reach the metaphase II (MII) stage [25]. Then, after fertilisation, only SN oocytes may develop to term, whereas NSN oocytes arrest at the 2-cell stage [25–28].

The two different nuclear phenotypes underlie specific transcriptional and translational programmes central to the acquisition of a correct developmental competence or developmental failure [29–32]. Their distinct chromatin organisations, which are found in most of the mammals studied (for a review see [33]), reflect the pattern of localisation and arrangements of the nucleolar organising region- (NOR-) bearing chromosomes and of the nuclear and perinucleolar localisation of centromeres and their associated pericentromeric CHC occurring during folliculogenesis [18, 19].

The crux of the present study is to contribute detailed information on the morphological transformations occurring to the nuclear organisation of the SN or NSN chromatin during meiosis resumption. We conducted a live observation of GV oocytes while progressing* in vitro* towards the MII stage, with the aim of giving an accurate description of the dynamics of this transition. To this end, we isolated fully grown antral oocytes from the ovaries of females primed with PMSG and, after staining with Ho, we performed a comprehensive 9 hr time-lapse imaging of both SN and NSN oocytes. The results describe a pathway of transition from the GV to the MII stage that is punctuated of discrete recordable events showing their specificity and occurring with different time kinetics in the two types of oocytes.

## 2. Materials and Methods

### 2.1. Animals

Four- to six-week-old CD1 female mice were used for this study (Charles River, Como, Italy). Mice were maintained by the Department of Animal Facility according to the Guide for Care and Use of Laboratory Animals, under conditions of 21°C temperature and a dark/light cycle of 12/12 hours. This research has been performed after the approval of the Animal Ethics Committee of the University of Pavia and carried out in strict accordance with the protocol approved by the European (n. 86/609/CEE) and Italian (n. 116/92, 8/94) legislation.

### 2.2. Hormonal Treatment

Forty-eight hours before sacrifice and oocytes isolation, female mice were intraperitoneally injected with 3.75 IU Folligon (PMSG, Intervet Italia, Segrate, Italy).

### 2.3. Isolation of Fully Grown Antral Oocytes

Fully-grown antral oocytes were collected in M2 medium by puncturing the ovarian surface with a sterile insulin needle. Then, they were freed from surrounding cumulus cells by gently pipetting in and out with a mouth-controlled hand-pulled glass Pasteur micropipette.

### 2.4. Classification and Maturation of Antral Oocytes to the MII Stage

Immediately, after their isolation form the ovary, cumulus-free antral oocytes were transferred into 20 *μ*L droplets of Ho fluorochrome (0.05 *μ*g/mL; Sigma-Aldrich, Milano, Italy, cat. N. B2261) in M2 medium for 12 min at room temperature. Details on the method of classification of SN or NSN oocytes are given in [24]. Three-four oocytes were transferred in each of four 2 *μ*L *α*-MEM drops (Life Technologies, Monza, Italy, cat. N. M4526) placed at the centre of a 3.5 cm glass-bottom Petri dish (WillCo Wells B.V., Amsterdam, The Netherlands, cat. N. GWSt-3522) and covered with mineral oil (Sigma-Aldrich, cat. N. M8410). *α*-MEM was supplemented with 5% heat-inactivated foetal bovine serum (Life Technologies, cat. N. 10270106), 2 mM L-Glutamine (Life Technologies, cat. N. 25030), 5 mM Taurine (Sigma-Aldrich, cat. N. T0625), and 36 *μ*g/mL sodium pyruvate (Sigma-Aldrich, cat. N. P4562). Oocytes were* in vitro* matured (IVM) for a total of 15 hr inside a BioStation IM (Nikon, Torino, Italy) at 37°C, under a 5% CO_2_ humidified atmosphere. At the end of the culture period, first polar body (PBI) extrusion was assessed by monitoring oocytes under an Olympus SZX9 (Olympus, Milano, Italy) stereomicroscope.

### 2.5. Time-Lapse Analysis

Time-lapse analysis was performed over a 9 hr total recording. Bright field and fluorescence images of oocytes were taken at 8 min time intervals and for time-lapse segments of a maximum of 1.5–2 hr; this segmentation was decided after preliminary experiments indicating that an exposure to UV irradiation prolonged beyond the 2 hr could affect the oocyte maturation. To cover the whole 9 hr recording period, five time-lapse segments were designed: from 0 to 2 hr (12 SN and 7 NSN oocytes were recorded), from 2 to 3.5 hr (6 SN and 5 NSN oocytes), from 3.5 to 5 hr (10 SN and 12 NSN oocytes), from 5 to 7 hr (9 SN and 6 NSN oocytes), and from 7 to 9 hr (20 SN and 7 NSN oocytes), for a total of 57 SN and 37 NSN oocytes analysed. At each 8 min time interval, a 15 sections Z-stack, 4 *μ*m spaced, was arranged. In order to produce the movies, all the time-lapse images acquired with the BioStation during each time segment analysed were subsequently imported into the Image J software and then converted into a file  .avi (7 frame per second). The GV (nucleus) area was measured using the CellSens Dimension software (Olympus). Since the GV shape is never that of a circle, this software allows drawing a Region Of Interest (ROI) by connecting a number of points that are chosen by the operator and then it calculates its area (pixel^2^).

### 2.6. Statistics

Differences in the timing in which meiotic events occurred were evaluated with Student's *t*-test and Mann-Whitney *U*. Changes in the nuclear area were evaluated using Student's *t*-test. Data analysed with the SigmaStat 3.5 software were considered significantly different when *P* < 0.05.

## 3. Results

In this study we observed the morphological changes that occur to NSN and SN fully grown antral GV oocytes (bright field) and to the organisation of their Ho-positive chromatin (fluorescence) during the transition towards the MII stage. The details that are given hereafter are representative of the observations made for each of the time-lapse phases recorded of those oocytes that reached MII.

### 3.1. Time-Lapse Imaging from 0 to 120 Minutes

**Figure 1:**
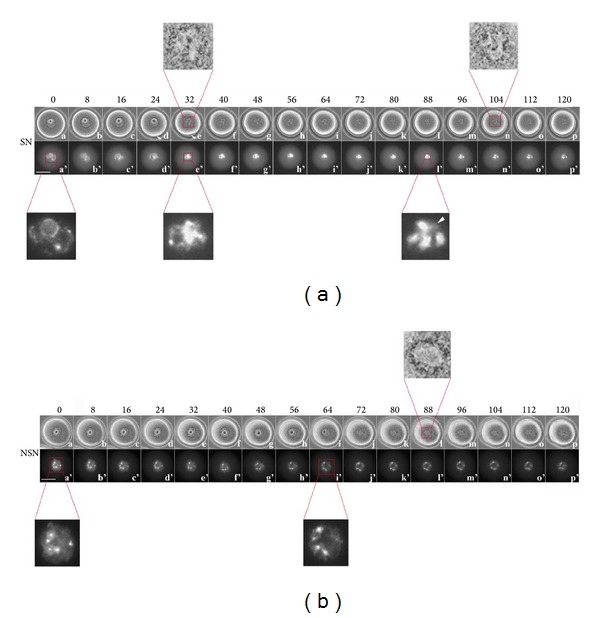
Time-lapse imaging during the first 120 minutes of oocyte culture. (a) SN oocyte, (a–p) bright field; (a'–p') UV fluorescence describing the changes to the Ho-positive chromatin within the nucleus. Arrow in (e) and (d) shows increase of perivitelline space. Arrowhead in (l') enlargement thread-like chromatin structures is visible among the pericentromeric CHCs. (b) NSN oocyte, (a–p) bright field; (a'–p') UV fluorescence. Bar: 40 *μ*m.

During the first 2 hr of SN oocytes culture, bright-field observations showed that the germinal vesicle break down (GVBD) occurs on average 34.2 ± 8.1 min since the starting of the recording (Table 1). GVBD is preceded by a 12.4% reduction (*P* < 0.01) of the GV area (Figure 1(a) (a–d)) and culminates with the disassembly of the nuclear envelope and dismantle of the characteristic rounded shape (Figure 1(a) (e); see Additional file 1 available online at http://dx.doi.org/10.1155/2014/207357). Around the nucleus we observed a thin and dark rim which assumes a granular pattern, that is, small black dots that first gather along the perimeter and then, with the disassembly of the nuclear envelope, move towards the nucleus area centre that remains visible, throughout the 2 hr observation, as a hollow (nuclear hollow, NH) (Figure 1(a) (e–n) and enlargements).

Almost coincidentally with the formation of the NH (24.0 ± 10.6 min), we observed a sudden detachment of the zona pellucida (ZP) at one side of the oocyte (Table 1; Figure 1(a) (d), (e), arrow; Additional file 1), likely due to a slight contraction. As a consequence, the size of the perivitelline space increases and is maintained as such throughout the remaining culture period. Whilst the increase of the perivitelline space was a feature of the gametes at this stage of maturation, in three out of twelve oocytes analysed we did not record this characteristic.

When observed under ultraviolet (UV) light, the typical SN chromatin configuration (Figure 1(a) (a') and enlargement) is maintained unaltered during the first 29.1 ± 14.0 min (Figure 1(a) (a'–d')); then, abruptly during the following 8 min and concomitantly with the GVBD (Figure 1(a) (e') and enlargement), we recorded an increased chromatin condensation in the area where the nucleolus was positioned (Additional file 2). During the remaining culture period, with the disappearance of the ring of Ho-positive chromatin, 4-5 large CHCs become visible (92.4 ± 9.9 min) (Table 1) (Figure 1(a) (l'–p')), with the exception of few thread-like chromatin structures visible among the CHCs (Figure 1(a) (l'), arrowhead in enlargement).

In NSN oocytes (Figure 1(b) (a') and enlargement), the features described above displayed overall more variable patterns. The GVBD occurred at 51.4 ± 19.0 min since the beginning of the culture period (Table 1; Figure 1(b) (j)), significantly later (*P* < 0.05) than that of SN oocytes, although in one oocyte it occurred at 16 min and in another occurred as late as 72 min. The rim of small black dots coalesced towards the centre of the NH at later stages of maturation compared to SN oocytes (Figure 1(b) (l) and enlargement). The size of the perivitelline space increased, but much later (96.0 ± 24.0 min; Table 1) (*P* < 0.001) compared to SN oocytes, and only in three out of seven oocytes analysed.

Under the UV light, throughout the 0 to 120 min interval, we recorded an almost static image showing the presence of 4/5 small Ho-positive CHC around the nucleolus (Figure 1(b) (a'–p'); Additional file 3). A visible change was observed at the time of GVBD when, at the disappearance of the nucleolus, the CHCs slightly moved towards the periphery (Figure 1(b) (i') and enlargement).

### 3.2. Time-Lapse Imaging from 128 to 216 Minutes

The size and shape of SN oocytes during this time-lapse period remained mainly unchanged and, at the bright field, we did not observe specific marker features.

**Figure 2:**
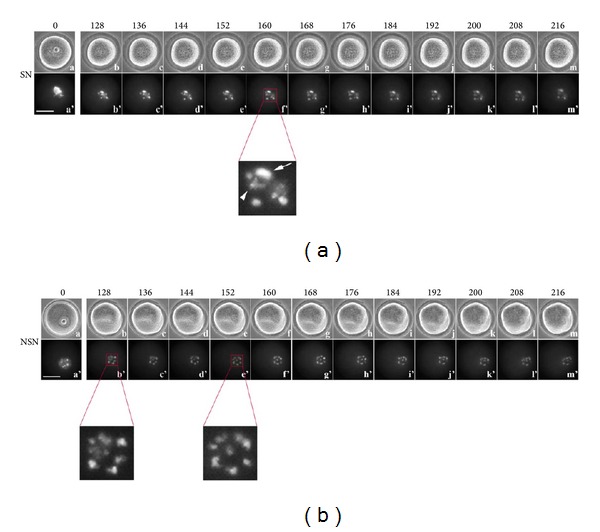
Time-lapse imaging from 128 to 216 minutes of oocyte culture. (a) SN oocyte, (a–m) bright field; (a'–m') UV fluorescence. Arrow in (f') enlargement, large CHC; arrowhead, small CHC. (b) NSN oocyte, (a–m) bright field; (a'–m') UV fluorescence. Bar: 40 *μ*m.

Under UV light, from the 128 min onwards, we recorded a progressive partial decondensation of the CHCs (Figure 2(a) (b'–m'); see enlargement: arrow, large CHC; arrowhead, small CHC; Additional file 4). In NSN oocytes, following the first 120 min of culture when chromatin organisation does not change, chromosomes appear abruptly (139.2 ± 20.9 min of culture (Table 1; Figure 2(b) (b') and enlargement), much earlier than in SN oocytes (see below) and assume a “rosette-like” organisation (Figure 2(b) (e') and enlargement) that is maintained up to the end of this recording time segment (Additional file 5).

### 3.3. Time-Lapse Imaging from 224 to 296 Minutes

**Figure 3:**
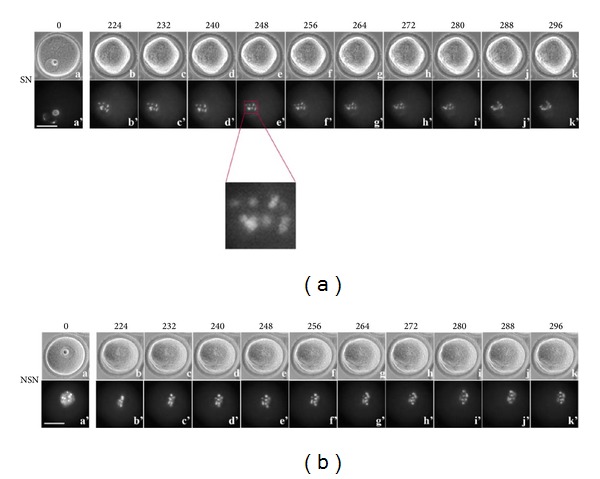
Time-lapse imaging from 224 to 296 minutes of oocyte culture. (a) SN oocyte, (a–k) bright field; (a'–k') UV fluorescence. (b) NSN oocyte, (a–k) bright field; (a'–k') UV fluorescence. Bar: 40 *μ*m.

In SN oocytes, 8–10 Ho-positive regions arrange to form a “rosette-like” distribution after 223.2 ± 20.8 min culture (Table 1; Figure 3(a) (b'–k') and enlargement), later (*P* < 0.01) than NSN oocytes. This organisation is maintained throughout this recording time segment (Additional file 6).

Chromosomes of NSN oocytes maintain the “rosette-like” disposition acquired during almost the whole period (Figure 3(b) (b'–k')), although, by the end, they begin moving towards the oocyte surface (Additional file 7).

### 3.4. Time-Lapse Imaging from 304 to 416 Minutes

**Figure 4:**
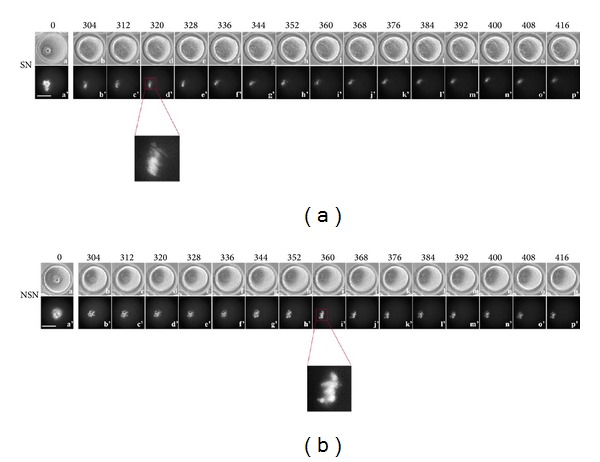
Time-lapse imaging from 304 to 416 minutes of oocyte culture. (a) SN oocyte, (a–p) bright field; (a'–p') UV fluorescence. (b) NSN oocyte, (a–p) bright field; (a'–p') UV fluorescence. Bar: 40 *μ*m.

This time-lapse segment is characterised by the formation of a clear MI plate, which appears earlier (*P* < 0.05) in SN (350.2 ± 34.6 min, Figure 4(a) (d') and enlargement) than NSN (390.7 ± 20.5 min, Figure 4(b) (i') and enlargement) oocytes. The MI plate changes localisation in both SN (Additional file 8) and NSN (Additional file 9) oocytes (Figure 4(a) (d'–p') and Figure 4(b) (i'–p')).

### 3.5. Time-Lapse Imaging from 424 to 536 Minutes

**Figure 5:**
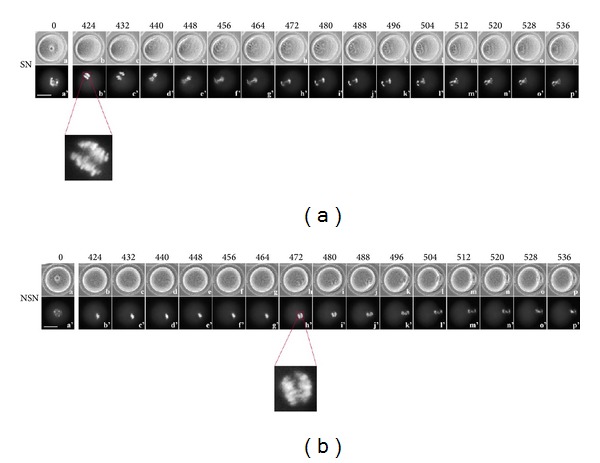
Time-lapse imaging from 424 to 536 minutes of oocyte culture. (a) SN oocyte, (a–p) bright field; (a'–p') UV fluorescence. (b) NSN oocyte, (a–p) bright field; (a'–p') UV fluorescence. Bar: 40 *μ*m.

Anaphase I (Table 1; Figure 5(a) (b'), Figure 5(b) (h') and enlargements), with two separated chromosome sets and PBI extrusion (SN: Table 1; Figure 5(a) (h)) (Additional files 10 and 11), is the main feature of this time-lapse segment. Although all the SN or NSN oocytes analysed reached the MII phase, the latter was attained before the end of the 9 hr recording period (480 min) by 55.0% SN or 14.3% NSN oocytes, respectively. The remaining 45.0% or 85.7% SN or NSN, respectively, attained the MII after the 9 hr of time-lapse recording, within the 15 hr of IVM.

## 4. Discussion

In its reductionist layout, the experimental application of the SN/NSN model is a powerful tool that allows having, at one's disposal, ovarian oocytes of known developmental competence or incompetence. Staining of the chromatin with the fluorochrome Ho gives the opportunity to identify within a pool of oocytes isolated from the antral compartment of the ovary those that may develop to term (SN) from those that certainly arrest development soon after fertilisation (NSN) [25–28]. This early classification allowed us to focus on emerging differences between developmentally competent and incompetent oocytes, while they are maturing* in vitro* from the GV to the MII stage. A first difference that emerges is the significant longer time, almost doubled, that the NSN oocytes spend at the diplotene stage before undergoing GVBD (NSN: ~51 min versus SN: ~34 min). When GVBD is almost completed and the nuclear envelope is dismantled, we observe a rim of black dots, seen under bright field in both types of oocytes, that represents mitochondria clearly visible for their autofluorescence when analysed at 440–490 nm wavelength ([34, 35]; our unpublished observations). Then, by the time the oocyte reaches the MII phase, these mitochondria disperse within the ooplasm and become invisible when observed at the bright field ([34, 35]; our unpublished observations). These early events in meiosis resumption are accompanied by significant rearrangements of the cytoskeleton [36] that we could clearly record in SN, but less extensively in NSN oocytes, as a pulse contraction of the gamete itself. This shrinkage brings, as a consequence, to an enlargement of the perivitelline space on the one side of the oocyte, increase that is maintained as such throughout the remaining culture period.

The typical chromatin organisation of SN and NSN oocytes is maintained unaltered and distinct during the whole diplotene stage. Then, coincidentally with the GVBD and the beginning of diakinesis, the SN chromatin undergoes numerous changes, instead the NSN chromatin preserves its original organisation for much further (up ~140 min). The NSN nucleus maintains a steady chromatin organisation with 4/5 small Ho-positive CHCs around the nucleolus, corresponding to the pericentromeric area of NOR-bearing chromosomes [18]. Instead, the SN chromatin abruptly condenses (~30 min) around the nucleolar area; then, 4/5 CHCs emerge and become larger in size and more separated one from the other, marking the end of diakinesis and the beginning of the following prometaphase (~100–120 min) [37]. The formation of the CHCs in SN oocytes has been explained with the gathering around the nucleolus of the pericentromeric regions of the 40 telocentric chromosomes of the mouse karyotype [18]. Then, these large CHCs become smaller in size and increase in number, likely as a consequence of the drifting away of chromosomes that later will begin to appear clearly visible as single entities arranged in a “rosette-like” organisation, a disposition of the chromosomes that marks the passage towards the MI phase.

Although these results show that the rosette-like figure is detected ~80 min earlier in NSN compared to SN oocytes, the transition to MI occurs earlier in SN (~350 min) compared to NSN (~390 min) oocytes, suggesting a longer permanence in prometaphase for the latter gametes. The extended prometaphase in NSN oocytes may be explained with chromosome lagging in the congression towards the MI plate formation and may be causal to the about 4-fold higher aneuploidy rate that we described in NSN compared to SN oocytes in females that have undergone the same hormonal treatment [38]. The correlation between chromosome lagging and aneuploidy will be further analysed with a more detailed time-lapse analysis of the first meiotic division.

In summary, NSN oocytes undergo chromatin changes, distinct from those of SN oocytes, which prepare the genome to accomplish the following meiotic phases and reach MII. We observed a longer GV-to-MII transition in NSN oocytes that reach the M-phase without the characteristic gathering of heterochromatin regions around the nucleolus [23]. Although we cannot identify a specific cause for the observed delay, a number of features that have been described may build up to a comprehensive picture of the biological nature of these two different antral oocytes. Interestingly, they display a different epigenetics status; that is, the SN chromatin configuration has higher levels of CpG methylation, histone acetylation (H4K5ac and H4K12ac), and methylation (H3K9me2) [39] which may be crucial to the dynamics of the large scale chromatin remodelling occurring soon after meiosis resumption. Interestingly, delayed transition was also described in SN oocytes treated with the histone deacetylase inhibitor trichostatin A [40], which prevents the onset of the global deacetylation occurring soon after meiosis resumption, indicating that perhaps NSN oocytes may present a lower or even damaged deacetylation activity. In addition to these described differences, our own whole transcriptome microarrays studies show that NSN oocytes exhibit upregulation of transcriptional networks associated with mitochondrial dysfunction and apoptosis and downregulation of cell cycle transcripts [29].

When considered together with a number of recent molecular data, our morphological observations help to further understand the biological significance of these two types of oocytes within the ovary. Morphological and molecular data speak in favour of a separation of the maturation pathways of SN and NSN oocytes and possibly a distinct fate within the ovary. Microarrays studies demonstrated the presence in SN oocytes of a transcriptional network (TN) regulated by the oocyte-specific transcription factor OCT4 (OCT4-TN), whose downregulation in NSN oocytes plays a key function in a sequence of molecular events that lead to their developmental arrest [29]. From these studies, OCT4 emerges as a crucial regulator of the events that govern the establishment of the developmental competence of mouse oocytes [29, 31]. Mostly important is the pattern of expression of OCT4, which remains confined to oocytes with an SN type of chromatin configuration from the beginning to the end of oocyte growth, whereas it is downregulated in NSN oocytes throughout folliculogenesis [30]. A similar profile of expression is shown by other OCT4-regulated genes, including STELLA (DPPA3) [30], another oocyte-specific transcription factor whose lack of expression leads to a developmental arrest mainly at the 2-cell stage [30, 41].

## 5. Conclusions

In this study, we have minutely described and filmed, using time-lapse imaging, modifications to the oocyte morphology and to its chromatin organisation. The overall picture that comes to light is that of a pathway of transition from GV to MII for the two types of oocytes which is punctuated of discrete recordable events that show their specificity and occur with different time kinetics. The main significant differences recorded during oocyte maturation are (1) a reduction of the nuclear area that occurs before the GVBD, significant only for SN oocytes; (2) a ~17 min delay of the GVBD in NSN oocytes; (3) an increased chromatin condensation, soon after the GVBD, that occurs only in SN oocytes; (4) the formation of 4-5 CHCs only in SN oocytes; (5) an increase of the perivitelline space that occurs ~57 min later in NSN oocytes; (6) the formation of a rosette-like disposition of the pericentromeric regions that takes place ~84 min later in SN oocytes; (7) the MI plate appears ~40 min later in NSN oocytes.

Altogether, morphological and molecular data of earlier studies build up to a model of mammalian ovary that envisages the coexistence of follicles enclosing oocytes that are potentially developmentally competent or incompetent and both capable of growth and meiotic differentiation, at least* in vitro*. This model raises numerous questions, including whether growing follicles containing developmentally incompetent NSN oocytes may be rescued, that is, guided to acquire the SN chromatin organisation and whether this is paralleled by the acquisition of a developmental competent state. The answer to this question would improve our understanding of the yet poorly known biology of the mammalian ovary and would carry positive clinical implications to enhanced assisted reproductive technologies.

## Supplementary Material

Additional files show the main events that happen throughout meiotic resumption (from 0 to 536 min) of mouse antral oocyte, both in developmentally competent (SN) or incompetent (NSN) oocytes. Time-lapse movies in bright field (Additional files 1 and 10) show changes in the nucleus, zona pellucida, perivitelline space and cytoplasm of the oocyte. Time-lapse movies under UV light (Additional files 2-9 and 11) show the changes that oocyte chromatin undergoes following GVBD and during the formation and segregation of chromosomes.

## Figures and Tables

**Table 1:** Timing of events occurring during meiosis resumption of antral oocytes cultured *in vitro*.

Type of event	Mean ± S.D. in minutes	NSN oocytes	*P* value*
	SN oocytes		
GVBD	34.2 ± 8.1	51.4 ± 19.0	<0.05
Increase of the perivitelline space	24.0 ± 10.6	96.0 ± 24.0	<0.001
Formation of 4-5 CHCs	92.4 ± 9.9	ND**	—
Formation of 8–10 small pericentromeric regions (rosette)	223.2 ± 20.8	139.2 ± 20.9	<0.01
MI plate	350.2 ± 34.6	390.7 ± 20.5	<0.05
Anaphase I	483.6 ± 33.0***	ND	—
Extrusion of polar body I and formation of MII plate	520.7 ± 20.7***	ND	—

*Comparison of SN versus NSN.

**Not detected.

***Calculated on 55% of oocytes that reached anaphase I within the 9 hr recording period.
